# Evidence-based intervention sustainability strategies: a systematic review

**DOI:** 10.1186/s13012-019-0910-6

**Published:** 2019-06-06

**Authors:** Maji Hailemariam, Tatiana Bustos, Barrett Montgomery, Rolando Barajas, Luther B. Evans, Amy Drahota

**Affiliations:** 1 College of Human Medicine, Division of Public Health, Michigan State University, Flint, MI USA; 2 Department of Psychology, Michigan State University, East Lansing, MI USA; 3 Community-Based Organizational Partners (CBOP), Flint, MI USA; 4 Child & Adolescent Services Research Center (CASRC), San Diego, CA USA

**Keywords:** Sustainment, Sustainability, Evidence-based interventions, Sustainment outcomes

## Abstract

**Background:**

Sustainability of evidence-based interventions (EBI) remains a challenge for public health community-based institutions. The conceptual definition of sustainment is not universally agreed upon by researchers and practitioners, and strategies utilized to facilitate sustainment of EBI are not consistently reported in published literature. Given these limitations in the field, a systematic review was conducted to summarize the existing evidence supporting discrete sustainment strategies for public health EBIs and facilitating and hindering factors of sustainment.

**Methods:**

We searched PsychINFO, Embase, ProQuest, PubMed, and Google Scholar. The initial search was run in March 2017 and an update was done in March 2019. Study eligibility criteria included (a) public health evidence-based interventions, (b) conducted in the community or community-based settings, and (c) reported outcomes related to EBI sustainment. Details characterizing the study setting, design, target population, and type of EBI sustained were extracted.

**Results:**

A total of 26 articles published from 2004 to 2019 were eligible for data extraction. Overall, the importance of sustainability was acknowledged across all of the studies. However, only seven studies presented a conceptual definition of sustainment explicitly within the text. Six of the included studies reported specific sustainment strategies that were used to facilitate the sustainment of EBI. Only three of the studies reported their activities related to sustainment by referencing a sustainment framework. Multiple facilitators (i.e., adaptation/alignment, funding) and barriers (i.e., limited funding, limited resources) were identified as influencing EBI sustainment. The majority (*n* = 20) of the studies were conducted in high-income countries. Studies from low-income countries were mostly naturalistic. All of the studies from low-income countries reported lack of funding as a hindrance to sustainment.

**Implication for dissemination and implementation research:**

Literature focused on sustainment of public health EBIs should present an explicit definition of the concept. Better reporting of the framework utilized, steps followed, and adaptations made to sustain the intervention might contribute to standardizing and developing the concept. Moreover, encouraging longitudinal dissemination and implementation (D&I) research especially in low-income countries might help strengthen D&I research capacity in public health settings.

## Background

Sustaining the changes that result from evidence-based public health interventions has become a topic of great interest among many researchers, donors, practitioners, and communities [1]. Evidence-based interventions (EBI) are defined as practices by which the provider’s decision is backed by the most appropriate information [2]. EBIs originated from the evidence-based medicine movement. In recent years, additional fields that involve routine intervention and clinical decision making have embraced this movement [3]. This includes a range of EBIs in treatment research, prevention, policy, medicine, community-based public health, and overall healthcare [4–7]. Although EBIs are conceptually appealing, our understanding of the implementation processes, including sustainment, that are necessary for delivering these practices over time in community-based settings remains unclear [1]. The field of dissemination and implementation (D&I) science has provided definitions for conceptually distinct terms, sustainability, and sustainment. Sustainability is defined as “the extent to which an evidence-based intervention can deliver its intended benefits over an extended period of time after external support… is terminated” [8] (p. 26), whereas sustainment is defined as “creating and supporting the structures and processes that will allow an implemented innovation to be maintained in a system or organization” [9].

### Research-to-practice gap

The past few decades have marked a significant shift from traditional diffusion of interventions and research outcomes—“passive, untargeted, unplanned, and uncontrolled spread of new interventions” [10]—to a more structured approach of EBI dissemination, implementation, and sustainment in order to reduce the oft-noted research-to-practice gap [11]. Current estimates suggest that it takes about 17 years to implement only 14% of evidence-based research outcomes in real-world settings [12, 13]. This research-to-practice gap often translates to suboptimal care for patients, exposure to potentially avoidable harm, excessive healthcare spending, and other significant opportunity costs [14]. Multiple and mutually interacting factors are believed to contribute to this large research translation gap. Previous studies report that inadequate training, limited time, lack of infrastructure, and lack of feedback and incentives for the utilization of EBIs hinder timely adoption and sustainment of EBIs in real-world settings [15]. Further, of the EBIs that are adopted and implemented, many EBIs are not sustained after a certain amount of time [15].

### Public health evidence-based interventions

Traditionally, the goal of intervention developers is to test the efficacy of the novel intervention in ideal settings with ideal participants and practitioners; however, this is not the typical end-point for public health researchers. For example, effectiveness research focuses on testing an individual-level intervention that is being delivered within “real-world” settings by usual care providers to community patients. The outcome measures still remain at the individual or family level [8]. However, sustainment of EBIs within community-based organizations requires the evaluation of the processes and factors that may facilitate or hinder the continuation of an EBI [9]. For example, the field of health system quality improvement emphasizes the comparative assessment of the value of organizational or system-level interventions that support the sustainability of EBIs [16]. Thus, careful planning is necessary to ensure swift and sustained applications of findings from evidence-based research into real-world settings [17]. Implementation studies in public health have emphasized the importance of developing sustainability strategies throughout the planning phase of an EBI to attain its utmost benefits at the individual, client, and organizational level [18]. Yet, the sustainment of EBIs in community-based settings remains a major challenge [19].

In the past few years, sustainability has been a buzz word in various disciplines, including public health research. Despite its appeal, the concept has remained undefined or loosely defined in most public health research, leading to underreported or vague findings [20]. The ambiguity in the conceptual and operational definition of sustainment has been identified as one of the barriers contributing to the large translational gap in public health and healthcare in general [21].

Sustainment of EBIs presents many complexities. While planning for the sustainment of EBIs, researchers and practitioners often face different unanticipated challenges pertaining to intervention characteristics, the organizational setting, and the broader policy environment [22]. For example, by overcoming these inner and outer contextual challenges, some public health interventions have been successfully sustained with fidelity. However, other EBIs may require adaptations over time to continue to work effectively in a complicated and dynamic real-world context [23, 24]. Effective sustainment strategies, outside of fidelity monitoring, for adapted interventions, are not well reported upon in implementation studies.

### Sustainment efforts in low- and middle-income countries

Challenges to implementing EBIs in communities within the US also carry over to low-income countries, where cultural differences, barriers to cost, accessibility and  quality are common. Given these barriers in adoption and dissemination of EBIs in low-income countries, sustaining any program efforts remains difficult [25].

The limited evidence from low- and middle-income countries (LMICs) reported that complexity of the intervention and inadequate program familiarity, limited human resource, and lack of workplace support for the new program and high staff turnover as barriers to sustainment [26–28]. Moreover, limited health system capacity, poor application of evidence-based interventions, inadequate involvement of local implementers, and high staff turnover also were reported to hinder the use of public health EBIs [28]. However, limited evidence exists regarding the sustainment of EBIs in LMICs compared to high-income countries (HICs) [29]. The broader factors influencing the sustainment of EBIs in these settings remain underreported.

Given that little is known about how and under what conditions sustainability occurs, it remains unclear as to what strategies facilitate or hinder sustainability outcomes [30]. For instance, strategies to facilitate maintenance of health benefits activities or workforce capacity have not been recognized because of the limited understanding to the developing process of sustainability [31, 32]. Therefore, there is a need to identify and describe existing facilitators or barriers to sustainment outcomes to better understand implementation processes, promote the use of impactful EBIs, and advance the field of dissemination and implementation science.

### Objectives

The goal of this systematic review was to understand the state of the literature related to the sustainment of public health EBIs. Specifically, we aimed to (1) describe how sustainability was defined; (2) identify and describe evidence-based sustainment strategies utilized in peer-reviewed public health literature; (3) identify methods for evaluating sustainment outcomes; (4) identify sustainment strategies utilized and with which specific stakeholder groups; (5) identify and describe reported sustainment outcomes; (6) develop recommendations for (a) reporting sustainment efforts as well as (b) utilizing specific sustainment strategies with specific stakeholder groups, both initially and when sustainment seems to be failing; and (7) sustainment efforts in low -income settings.

## Methods

Methods for the systematic followed the Preferred Reporting Items for Systematic Reviews and Meta-Analyses (PRISMA) guideline. We specified the methods in advance and documented every step in an a priori protocol. The protocol was updated iteratively throughout the systematic review (the systematic review protocol is available upon request from the first author).

### Eligibility criteria

Eligible studies included articles that (1) were peer-reviewed, (2) written in the English language, (3) reported the use of a specific EBI, (4) involved the implementation of an EBI in a community-based setting, and (5) provided a description of strategies used to sustain the EBI. Our search was not restricted to specific population or year of publication. We excluded articles that were (1) not based on original data, (2) generic reports that did not focus on a specific EBI, or (3) reviews of other published or unpublished evidence. We did not include unpublished (ongoing) studies because these were not available on the databases we searched.

### Information sources

Multiple electronic database indexes were searched for potentially eligible articles, including PsycINFO (1887–present), ProQuest including ERIC and CSA social sciences’ abstracts (1971–present), PubMed (1946–present), Embase (1976-present), and Google Scholar (2004–present). Further, experts in the area of EBI sustainment were contacted by the senior author and asked to suggest additional relevant articles that had not been included. Finally, reference lists of identified articles (including systematic reviews) were checked for potentially eligible articles. Duplicate articles were excluded at each stage of the search process. We conducted an initial search in March 2017, and additional time-restricted searches (March 2017–March 2019) were run to identify more recently published studies.

### Search strategy

The following keywords were searched in any combination: “sustainment,” “sustainability,” “scale-up,” “continuation,” “health,” “providers,” “community,” “policy,” “services,” and “interventions.” These search terms were identified during a preliminary search of the literature focused on discovering the various terms used in articles related to the sustainment of EBIs. A filter was used in all searches to exclude review articles, articles in other languages, or articles that are not peer-reviewed.

### Study selection

The first and second authors reviewed the titles and abstracts identified by the searches. Articles were eligible for the full-text review if the title or abstract referenced as follows: (1) use of any specified EBI, (2) if the EBI was delivered in a community-based organization, and (3) provided a description of sustainment process. If project members could not determine initial eligibility from the title and abstract, the article passed to the next stage for a full-text review. Articles were excluded if the title and abstract did not pertain to EBI sustainment.

Two project members independently reviewed all of 4892 titles screened for inclusion. Inter-rater agreement for inclusion between the independent coders was 86%. Disagreements between reviewers were resolved with discussions aimed to develop a consensus about the eligibility of studies, with consultation from other co-authors, as needed.

After the title and abstract review, members of the coding team were randomly assigned articles (10–15 articles each) to review. Two project members independently reviewed the full-text of each article to determine inclusion into or exclusion from the systematic review. Disagreements were resolved through discussion between the two reviewers and a third independent reviewer until consensus was reached.

### Data collection process

A data extraction form containing an initial coding scheme was developed a priori, based on the study objectives and preliminary conceptualizations of sustainment strategies. Additional codes were generated deductively by the data extraction team, which consisted of four reviewers. Training for the reviewers included a half-day didactic coding workshop involving an introduction to the PRISMA statement [33] and discussion of each variable definition using practice articles. Raters reconvened to review how a second practice article was independently rated by each of the reviewers and to resolve any discrepancies or ambiguities about the coding process. All raters were asked to independently code and review four additional articles to ensure clarity of the variables and consistency in the coding process. Raters were provided coding documents containing assignment sheets, training slides and notes, the survey, variable operational definitions, and printed articles for review. Twelve consensus meetings were held during the initial full-text review. The coding template was further refined for the next level of the review. The new template was pilot-tested with 10% of the articles, discussed, and endorsed to be used for the final full-text review. Additional 10 consensus meetings were held for the final full-text review.

### Data items

The extracted data comprised of 45 items focusing on (1) basic publication details about the article (i.e., publication date, author), (2) study design and methods, (3) reported EBI outcomes, (4) sustainment strategies, (5) targeted audience of sustainment strategies (e.g., organizational leaders, direct providers), (6) barriers and facilitators influencing sustainment outcomes, and (7) miscellaneous details on funding and comments/concerns about the articles. Information about the data items is available in the systematic review protocol.

### Risk of bias

To establish the strength of the body of evidence, we evaluated the risk of bias in individual studies and across studies. In individual studies, we checked validity and reliability of the measures used, study setting, appropriateness of the study design, methods of data collection, and how these interacted with reporting and outcomes [34]. We did not restrict our search to outcomes (positive/negative) to minimize the possibility of publication bias [35].

### Data analysis

All of the articles were independently double-coded by pairs of raters. Whenever disagreements emerged, the group of coders met to discuss coding disagreements until consensus was reached. All data were collected using the Qualtrics online survey program [36]. Raters received a link to the online survey via email. All coding was first conducted on printed articles and then entered into the online survey to create the database. Data were later exported to SPSS V.14 [37] for analysis. The review team (MH, TB, RB, and BM) met to discuss emerging themes and to create a reporting structure based on the objectives of the study. Recurrent themes that were identified were thematically categorized to facilitate reporting. Moreover, we also compared studies from high-income countries and low- and middle-income countries (LMICs).

## Results

### Study selection

**Fig. 1:**
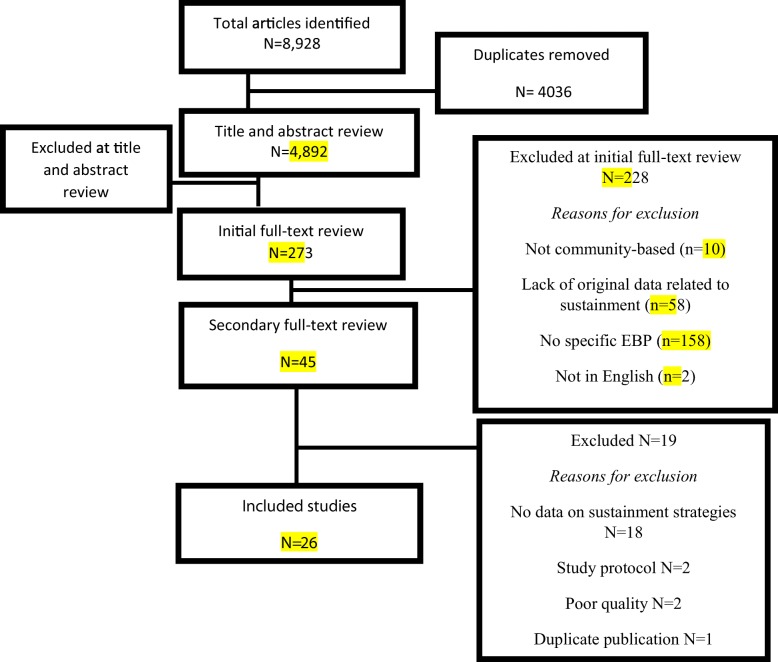
Flow chart of the study

Searches generated 4892 articles published up to March 2019 (Fig. 1). After reviewing these articles’ titles and abstracts, 274 articles were determined to meet the criteria for a full-text review. After the initial full-text review, 45 articles met the criteria for final inclusion. During data extraction, 19 articles were excluded because they did not fit the criteria of containing original data about sustainment strategies (*n* = 17) or because they were a study protocol or conceptual paper (*n* = 2), were of poor quality based on analysis of rigor and risk of bias (*n* = 2), or were a duplicate publication (*n* = 1).

#### Characteristics of the studies

A total of 26 articles published from 2004 to 2019 were included in the final analysis. The study settings include health care facilities (*n* = 7), other community-based organizations (*n* = 6), and communities (*n* = 13). The EBIs being sustained covered a variety of topics including physical health, behavioral health, prevention services, and life skills (Table 1).

**Table 1:** Types of EBIs included in the review

EBI	Topic	Frequency (*n*)
Active Living by Design (ALbD)	Physical health/health care	*n* = 10
Community-Based Reproductive Health Project		
Disease Management Programs		
Eye Care Program		
Hospital Elder Life Program (HELP)		
Innovations in Clinical Genetics		
Nurse-Initiated HIV Rapid Testing		
Putting the P.I.E.C.E.S. Together		
The Healthy Learners Asthma Initiative		
Actively Changing Together	Behavioral health	*n* = 9
Adolescent Community Reinforcement Approach		
After-School Behavioral Program		
CBT		
Massachusetts Tobacco Control Program		
Postpartum Women Programs		
SafeCare Program		
Smoking Cessation Program		
Communities That Care	Prevention services	*n* = 3
The Cuban Ae. Aegypti Control Programme		
The Sonagachi Project		
Individual Placement and Support (IPS) Model of Supported	Life skills	*n* = 4
Employment		
The Early Risers Skills for Success		
The Partner Program		

#### Quality of included studies

We used the Strengthening the Reporting of Observational Studies in Epidemiology (STROBE) [38] to evaluate the quality of cross-sectional studies and the Consolidated Standards of Reporting Trials (CONSORT) guideline [39] for appraisal of RCTs. Overall, the studies included in this review rated from moderate to high. Study design, objectives, and sampling were clearly presented in all of the studies.

### Conceptualization of sustainability

In all of the studies, the importance of sustainability was acknowledged. However, only ten out of the 26 studies included an explicit definition of sustainability. For 16 of the 26 studies, sustainability was inadequately defined or was missing altogether. Specifically, these studies discussed outcomes and influences of sustainability without an explicit definition of sustainability referenced in the text. We reviewed the study objectives of these 16 studies to determine how sustainability was conceptualized. In 17 of the studies, sustainability was conceptualized in relation to the continuity of a program sustained after the implementation phase. The concept of sustainability in these studies varied; however, we mapped these conceptualizations onto a consolidated list of definitions developed by Moore and colleagues [40] (see Table 2).

**Table 2:** Defining sustainment

Definition	Number of studies
After a defined period of time, the program, clinical intervention, and/or implementation strategies continue to be delivered	*n* = 17
Individual behavior change (provider and patient level) is maintained continuing to produce benefits for individuals or systems	*n* = 4
No definition of sustainment provided	*n* = 2
Retention of interventionists and clinic staff (staff retention as a marker of sustainment)	*n* = 1
Maintenance of core treatment elements (by providers) following the end of the implementation support period	*n* = 1
Services are maintained after the termination of major financial, managerial, and technical assistance from an external donor	*n* = 1

The following provide examples of the variability in sustainment conceptualization across studies:

In the field of public health, sustainability has been defined as the capacity to maintain program services at a level that will provide ongoing prevention and treatment for a health problem after the termination of major financial, managerial, and technical assistance from an external donor. [41]

Another study used the definition of sustainability from Glasgow [42] at the individual and organizational level.

At the individual level, sustainability has been defined as the long-term effects of a program as assessed after 6 or more months following the most recent intervention contact. [43]

Further, a study from Ghana defined sustainability as the “continuation of benefits” [44]. While another study [45] conceptualized sustainment in terms of retaining the human capacity of service providers and service users:

… a program is sustained if it continues to employ staff, maintains an active client caseload, and provides direct services. Programs sometimes continue in name only, without adhering to the program model that they originally implemented. [45]

### Specific efforts focusing on sustainment

Of the 26 studies included, six studies [9, 46–50] reported purposefully building sustainment efforts into the EBI implementation. These studies reported the initiatives they took to ensure the sustainment of the program outcomes after the end of the implementation phase. Further, only five studies reported the use of a specific dissemination and implementation framework that guided the sustainment efforts (Table 3). Notably, the remaining 21 studies described their sustainment activities without making any reference to a known a priori theoretical model or framework.

**Table 3:** Use of sustainment frameworks

Name of the framework	Description	Number of studies
Exploration, Preparation, Implementation, Sustainment (EPIS) Framework	An iterative approach guiding implementation of EBPs	*n* = 1
Reach, Effectiveness, Adoption, Implementation, Maintenance (RE-AIM)	A reliable tool to assess the impact of EBPs in various settings	*n* = 1
Framework by Shediac-Rizkallah and Bone [32]	Categories for monitoring sustainability: (1) maintenance of health benefits achieved through an initial program, (2) level of institutionalization of a program within an organization, and (3) measures of capacity building in the recipient community The use of programmatic approaches and strategies that favor long-term program maintenance including (1) project design and implementation factors, (2) factors within the organizational setting, and (3) factors in the broader community environment	*n* = 1
Program Sustainability Assessment Tool (PSAT)	Developed by the Center for Public Health Systems Science (CPHSS) at Washington University, the PSAT assesses a program's sustainability capacity across the eight domains: (a) political support, (b) funding stability, (c) partnerships, (d) organizational capacity, (e) programme evaluation, (f) programme adaptation, (g) communications, and (h) strategic planning (Luke et al., 2014).	*n* = 2

### Sustainment strategies used

Sustainment strategies extracted for the systematic review were guided by the conceptual model of factors that influence the sustainment of EBIs [3]. Nine sustainment strategies were identified among the 26 articles (Table 4). Funding and/or contracting for EBIs continued use (*n* = 12) and maintenance of workforce skills through continued training, booster training sessions, supervision, and feedback (*n* = 9) were most frequently reported. Other sustainment strategies included organizational leader stakeholder prioritizing and supporting continued use (*n* = 6), agency priorities, and/or program needs are aligned with new EBI (*n* = 4), maintenance of staff buy-in (*n* = 3), accessing new or existing money to facilitate sustainment (*n* = 2), systematic adaptation of the EBI to increase continued fit/compatibility of the EBI with the organization (*n* = 8), mutual adaptation between the EBI and organization (e.g., adaptation of the EBI to improve fit and alignment of the organizations’ procedures) (*n* = 7), and monitoring EBI effectiveness (*n* = 2). Two of the remaining studies reported that a specific sustainment strategy was not used, and the final three studies described utilizing “positive implementation climate” and “community engagement/partnerships” as sustainment strategies.

**Table 4:** Sustainment strategies used to sustain EBIs

Factor	Frequency
Sustainment strategies (*n* = 9)	
Funding and/or contracting for EBIs continued use	*n* = 11
Maintenance of workforce skills through continued training, booster training sessions, supervision, and feedback	*n* = 8
Mutual adaptation between the EBI and organization (e.g., adaptation of the EBI to improve fit AND alignment of the organizations’ procedures)	*n* = 7
Systematic adaptation of the EBI to increase continued fit/compatibility of the EBP with the organization	*n* = 7
Organizational leader stakeholder prioritizing and supporting continued use	*n* = 5
Agency priorities and/or program needs are aligned with new EBI	*n* = 4
Maintenance of staff buy-in	*n* = 2
Other	*n* = 2
None specified	*n* = 2
Accessing new or existing money to facilitate sustainment	*n* = 1
Monitoring EBI effectiveness	*n* = 1

### Sustainment strategies and stakeholder groups

Of the 26 articles, only nine studies [46–54] provided information about the specific intended audience (e.g., stakeholder groups) of the sustainment efforts. Intended audiences for EBI sustainment efforts included direct providers (*n* = 6), supervisors of direct providers (*n* = 4), organizational leaders (*n* = 5), and service users (*n* = 3) (Table 5). No studies targeted policy-makers in their sustainment efforts.

**Table 5:** Target audience for EBI sustainment efforts

Study	Targeted audience for EBI sustainment efforts			
	Direct providers	Supervisors	Organizational leaders	Service users
Fagan, et al. [54]	X	X	X	X
Grow, et al. [47]	X			X
Lyon, et al. [49]	X			
Palinkas, et al. [50]	X			
Splett, et al. [48]	X			
Romani, et al. [46]	X	X	X	X
Bergmark, et al. [51]	X		X	
Smith, et al. [52]			X	
Llauradó, et al. [53]	X		X	

### Sustainment outcomes

Moreover, the nine articles that reported specific intended audiences for their sustainment efforts also provided details on outcomes related to their sustainment strategy use. These details were grouped into two categories: (1) sustainment outcomes related to the implementation process and (2) sustainability outcomes directly related to the EBI. For the first category, details on sustainment outcomes included the moderating role of leadership styles (*n* = 1), the importance of tracking program activities to ensure continued use (*n* = 1), increased rates in initial and continued use of the EBIs (*n* = 3), and assessments related to degrees of institutionalization of EBIs (*n* = 1). Sustainability outcomes directly related to the EBI included increased usage of EBI components maintained over time (*n* = 2) and increased individual-level outcomes (e.g., asthma medication use and education) from EBI use (*n* = 1).

### Facilitating and hindering factors of EBI sustainment

Twenty-six facilitating and 23 hindering factors were reported to be of influence on the sustainment of EBIs (Table 6). Utilizing the influences on sustainability framework [20], we mapped each reported facilitator and hindrance onto the framework, which proposes four major thematic areas, including (1) innovation characteristics, (2) context, (3) capacity, and (4) processes and interactions. Funding (*n* = 16), adaptation/alignment (*n* = 15) and organizational leadership (*n* = 12) were the most frequently reported facilitating factors for EBI sustainment. No or limited funding (*n* = 13) was the most frequently reported hindering factor for EBI sustainment.

**Table 6:** Facilitating and hindering factors, definitions, and frequencies

Factor	Influences on sustainability	Frequency
Facilitating factors (*n* = 26)		
Adaptation/alignment	Processes and interactions	*n* = 14
Funding	Capacity	*n* = 13
Organizational leadership	Context	*n* = 12
EBP fit (acceptability, accessibility, adequacy, and cultural appropriateness)	Innovation characteristics	*n* = 11
EBP effectiveness or benefit	Innovation characteristics	*n* = 11
Training and education	Processes and interactions	*n* = 11
Ongoing support	Processes and interactions	*n* = 11
Setting characteristics (structures, policies)	Context	*n* = 10
Community stakeholder support/involvement	Capacity	*n* = 10
Ability to modify/did modify the EBP	Innovation characteristics	*n* = 9
Workforce (staffing, staff attributes)	Capacity	*n* = 9
Collaboration/partnership	Processes and interactions	*n* = 9
Integration of rules and policies	Processes and interactions	*n* = 7
Evaluation and feedback	Processes and interactions	*n* = 6
Resources	Capacity	*n* = 6
System/policy change	Context	*n* = 5
Internal/external EBP champions	Capacity	*n* = 5
Organizational climate	Context	*n* = 5
Engagement/relationship building	Processes and interactions	*n* = 5
Shared decision making among stakeholders	Processes and interactions	*n* = 4
Planning	Processes and interactions	*n* = 4
Organizational culture	Context	*n* = 4
Ability to maintain EBP fidelity/integrity	Innovation characteristics	*n* = 3
Navigating competing demands	Processes and interactions	*n* = 1
Other	Processes and interactions	*n* = 5
No facilitators factors were reported		*n* = 0
Hindering factors (*n* = 23)		
No/limited funding; funding ended or eliminated	Capacity	*n* = 11
Lack of resources	Capacity	*n* = 7
No hindering factors were reported		*n* = 6
Unable to navigate competing demands	Processes and interactions	*n* = 6
Organizational leadership did not support the sustainment of EBP	Context	*n* = 5
Other	Lack of adequate number of service users	*n* = 5
Workforce (staffing, staff attributes)	Capacity	*n* = 5
Setting characteristics (structures, policies)	Context	*n* = 4
Community stakeholders did not support the sustainment of EBP	Capacity	*n* = 3
EBP effectiveness or benefit was not observed	Innovation characteristics	*n* = 3
Lack of trained personnel to continue the EBP	Capacity	*n* = 3
No ability to modify/did modify the EBP	Innovation characteristics	*n* = 3
Organizational climate did not support the sustainment of EBP	Context	*n* = 3
Training and education was not sustained	Processes and interactions	*n* = 3
EBP did not fit	Innovation characteristics	*n* = 2
No ongoing support	Processes and interactions	*n* = 2
No sustained planning	Processes and interactions	*n* = 2
Not able to maintain EBP fidelity/integrity	Innovation characteristics	*n* = 2
Poor collaboration/partnership	Processes and interactions	*n* = 2
Internal/external EBP champions did not support the sustainment of EBP	Capacity	*n* = 1
System/policy change	Context	*n* = 0

#### Studies from LMICs

Of the eligible studies, only five of them [44, 46, 55–57] were conducted in LMICs according to the World Bank classification of countries [58]. One study did not report the study setting. All of these studies from LMICs followed a naturalistic approach with no longitudinal or RCT design reported. All but one [46] of the studies from LMICs was conducted in a facility-based setting. Regarding barriers to sustainment, all of the studies from LMICs reported that EBIs were not sustained after the termination of funding. Moreover, no study from LMICs reported actual targets of sustainment strategies.

## Discussion

There is a growing interest to assess sustainment to promote EBIs in public health research. Despite this emerging emphasis, there remains a large research-to-practice gap [12, 13] that can be attributed to inconsistent definitions and underreporting of sustainability. To help address these gaps, this systematic review provides a detailed summary of the current evidence of sustainability in public health interventions across various community-based settings and populations. To our knowledge, this is the first comprehensive systematic review that summarized definitions of sustainment and evidence-based intervention sustainment strategies targeting specific audiences within public health literature.

Although the importance of sustainability was acknowledged across all the studies, the concept was inadequately defined with only seven studies presenting a definition of sustainability somewhere in the text. Only nine of the included studies reported their sustainment efforts [9, 46–50]. Even fewer studies [9, 46, 47, 52, 53] presented their activities related to sustainment by referencing a known sustainment framework.

Evidence exists that various public health interventions are successfully implemented in academic settings. Nevertheless, ensuring their transferability to community settings or community-based organizations while also maintaining fidelity has been a challenge [59, 60]. This challenge could be attributable to a lack of clarity or knowledge about the appropriate frameworks and the steps followed towards the sustainability of the intervention [61]. Consistent with these findings, in our systematic review, only five studies [9, 46, 47, 52, 53] reported a pre-existing framework used to ensure the sustainment of the EBI. Our findings support that studies evaluating sustainment strategies are limited. Therefore, it is believed that this underreporting may even further lengthen the research-practice gap.

Understanding factors related to the implementation of evidence-based public health interventions has been of significant scholarly attention in recent years [23, 62–64]. However, lack of proper conceptualization of sustainment from the outset seems to have cast a shadow over further development of the field. In majorities of the included studies, sustainment was equated with the continuation of a program or an intervention after a defined period of time [9, 43, 44, 49–53]. Although sustainment is widely acknowledged as relevant, consistent with what was reported before [20], efforts to explicitly define the concept have been found minimal in this systematic review, with only seven studies presenting a definition of sustainability somewhere in the text. This review underscores the apparent need for including a clear and unequivocal definition of the concept of sustainment in the context of public health evidence-based interventions.

Previous studies have identified multiple hindering factors to the sustainment of EBIs in community-based settings. These include lack of funding, leadership challenges, unfavorable organizational climate, nature of the EBI, inadequate technical assistance, and fidelity monitoring [65, 66]. In this systematic review, 19 studies reported hindering factors related to the sustainment of EBIs. The challenges reported in those studies were also consistent with what was previously reported. Integrating a built-in mechanism to address leadership challenges and tailoring technical assistance to provide community stakeholders with the tools to adapt EBIs with or without the EBI developers may be relevant. Moreover, equipping community-based organizations with the skills to identify potential funding sources that can support the continuation of the program after a certain period might be important for sustaining EBIs in these settings.

No RCTs or studies with the longitudinal design were identified from low-income countries. There remains a need for more knowledge regarding sustainment efforts of EBIs across LMICs. In part, this could be attributable to the expensive nature of designing and conducting RCTs in resource-constrained settings. Most LMICs generally have limited primary research, and most evidence-based interventions are resource intensive, requiring structural and financial provisions [67].

## Limitations

While the reported results carry important implications for public health research, we should consider limitations to our systematic review. The review only included studies of sustainability published in peer-reviewed literature. “Gray literature” and unpublished literature were excluded, presenting potential publication bias. We only reviewed studies focused on public health interventions. Studies related to contexts outside of public health interventions were not included, potentially overlooking further details to inform the concept of sustainability. Future research should consider reporting the sustainment of EBIs in settings outside public health interventions.

## Conclusions

Studies reporting sustainment-related outcomes might benefit from presenting an explicit definition of the concept from the outset. Better reporting of the steps followed, frameworks used and adaptations made to sustain the intervention might contribute to standardizing and developing the concept. Moreover, encouraging longitudinal D&I research especially in low-income countries might help strengthen D&I research capacity in these settings.

## Data Availability

Systematic review protocol is available from the first author up on request.

## Abbreviations

CONSORT: Consolidated Standards of Reporting Trials
D&I: Dissemination and implementation
EBI: Evidence-based interventions
HIC: High-income countries
LMICs: Low- and middle-income countries
PRISMA: Preferred Reporting Items for Systematic Reviews and Meta-Analyses
RCT: Randomized controlled trial
STROBE: Strengthening the Reporting of Observational Studies in Epidemiology
